# Co-production and Managing Uncertainty in Health Research Regulation: A Delphi Study

**DOI:** 10.1007/s10728-019-00383-9

**Published:** 2019-08-31

**Authors:** Isabel Fletcher, Stanislav Birko, Edward S. Dove, Graeme T. Laurie, Catriona McMillan, Emily Postan, Nayha Sethi, Annie Sorbie

**Affiliations:** 1 J. Kenyon Mason Institute for Medicine, Life Sciences and the Law, School of Law, University of Edinburgh, Old College, South Bridge, Edinburgh, EH8 9YL UK; 2 School of Public Health, University of Montreal, 7101, Avenue du Parc, 3e étage, Montréal, QC H3N 1X9 Canada; 3 J. Kenyon Mason Institute for Medicine, Life Sciences and the Law, Usher Institute, University of Edinburgh, 23 Buccleuch Place, Edinburgh, EH8 9LN UK

**Keywords:** Collaboration, Co-production, Health research regulation, Proportionality, Public interest, Regulatory stewardship, Stakeholders

## Abstract

European and international regulation of human health research is typified by a morass of interconnecting laws, diverse and divergent ethical frameworks, and national and transnational standards. There is also a tendency for legislators to regulate in silos—that is, in discrete fields of scientific activity without due regard to the need to make new knowledge as generalisable as possible. There are myriad challenges for the stakeholders—researchers and regulators alike—who attempt to navigate these landscapes. This Delphi study was undertaken in order to provide the first interdisciplinary and crosscutting analysis of health research regulation, as it is experienced by such stakeholders in the UK context. As well as reinforcing existing understandings of the regulatory environment, Delphi participants called for greater collaboration, and even co-production, of processes involved in health research regulation. On the basis of this research, we offer insights about how health research regulation can become a matter with which a wider range of stakeholders—including researchers, regulators, publics and research sponsors—can engage. The evidence supports the normative claim that health research regulation should continue to move away from strict, prescriptive rules-based approaches, and towards flexible principle-based regimes that allow researchers, regulators and publics to co-produce regulatory systems serving core principles. By unpacking thorny concepts and practices at the heart of health research regulation—including the public interest and public engagement—our results have the potential to situate and breathe life into them. The results also demonstrate that while proportionality is well-recognised as a crucial element of flexible regulatory systems, more must be done to operationalise this as an ethical assessment of the values and risks at stake at multiple junctures in the research trajectory. This is required if we are to move beyond proportionality as a mere risk-management tool. Compliance culture no longer accurately reflects the needs and expectations of researchers or regulators, nor does it necessarily produce the best research. Embracing uncertainty—both as a human practice and a regulatory objective—may represent the brighter future for health research.

**Electronic supplementary material:**

The online version of this article (10.1007/s10728-019-00383-9) contains supplementary material, which is available to authorized users.

## Introduction

The architecture of health research has vastly expanded over the past two decades, as has the range of actors involved. Today, health research integrates studies involving tissue, health, lifestyle and genetic/genomic data, metadata and social media content. These developments have resulted in a burgeoning of often siloed sets of ‘regulatory spaces’ [16, 31], which focus on the nature of the objects of research rather than the interests and objectives at stake. There is also an increasing blurring of the distinctions between traditional roles such as clinician or researcher and patient or participant, not to mention between research and clinical treatment. The focus of enquiry is in a broad field termed here ‘health research regulation’. This is not a formal term of art, but rather refers to the general ecosystem of activities, laws and regulations that seek to shape the conduct of any and all types of research involving human participants, or materials, data or tissues donated by them. This involves a complex morass of regulations and actors, albeit in the European context many legal requirements derive from European Union law, as is the case with clinical trials, medical devices, data protection, and advanced therapy medicinal products (ATMPs).

It is not uncommon in the realm of human health research for regulators to adopt the ‘command and control’ model of regulation, as is commonly understood in regulatory theory [4, 30]. This involves a centralised authority, usually wielding legal powers of inspection and sanction, to oversee an area of health research. Typical examples are clinical trials and reproductive research, as demonstrated by the extensive powers of the UK’s Medicines and Healthcare products Regulatory Agency and Human Fertilisation and Embryology Authority, respectively. However, as human health research has expanded rapidly in recent decades to involve more work with personal data and biological samples (e.g. blood and tissue), this model has not been replicated. Rather, more reliance has been placed on regulatory approaches such as consent or anonymisation to authorise research. In other words, provided a participant consents to their data and samples being used, or the data and samples have been anonymised rendering re-identification effectively impossible, research may proceed. These approaches, too, however, have met with critics both for inadequately protecting and respecting research participants and/or blocking important kinds of research [1, 2]. A recent report from the Academy of Medical Sciences [3] called for a holistic approach to health research in an attempt to tackle some of these challenges, but there remains a lack of evidence and insight as to what form this might best take. Accordingly, this Delphi study was motivated by a concern that regulation of health research in the UK and Europe currently may be failing effectively to protect and promote the core values and objective of achieving improvements in human health while also responding well to erosion of the traditional socio-legal distinctions noted above. This threatens the ability of biomedicine to deliver both individual and social benefit. At the same time, the study did not assume that more law or regulation is necessarily the answer.

The key goal of this Delphi study is to provide the first interdisciplinary and crosscutting analysis of health research regulation as it impacts on the realisation of the goals of health research. It has sought to capture the views and experiences of stakeholders—both regulators and researchers—regarding regulation in order to inform better policy and legal responses. It aims to expose any gaps between law and regulation, on the one hand, and research practice, on the other. It seeks to explore opportunities and barriers to move within and between regulatory spaces, and to throw light on areas of uncertainty and instability. This study captures evidence of health research regulation as directly experienced by key stakeholders who inhabit the ecosystem; this is set against the core concepts and approaches that have typified health research regulation to date, notably in the UK and European regulatory environments. While the majority of the Delphi participants work in the UK sector, it is notable that many laws and regulation on health research emanate from the European Union (Box 1). Accordingly, the results have a wider relevance for all stakeholders operating within such a regulatory ecosystem.

**Box 1:** Key legal, regulatory, social and political events occurring at the time of the Delphi study

The Care Act 2014, which established the Health Research Authority (HRA) as a non-departmental statutory body with responsibility for health and social care research governance
The failure of the ‘care.data’ patient data sharing regime and subsequent concerns regarding information governance in NHS England
UK implementation of revised Caldicott principles regarding information governance in the health sector, specifically the handling of patient-identifiable information
Drafting and subsequent implementation of new EU General Data Protection Regulation (Regulation EU 2016/679)
New EU regulation of clinical trials on medicinal products for human use (Regulation EU 536/2014)
New EU regulations of medical devices (Regulation EU 2017/745) and in vitro diagnostic medical devices (Regulation EU 2017/746)
Changes to HRA regulatory approvals system, namely HRA Approval, bringing together the assessment of governance and legal compliance
Brexit (i.e. the potential withdrawal of the United Kingdom from the European Union)

## Methods

### Study Design

The Delphi survey technique was chosen because its use of open-ended questions minimises bias introduced by the researchers, allowing the research agenda to be set by study participants themselves in an area where ‘appropriate historical/economic/technical data [is not available] and thus where some form of human judgmental input is necessary’ [26: 354]. As defined by standard Delphi methodology, the study consisted of an ‘interactive, sequential and multi-step’ characterisation of expert views relying on iterative rounds allowing (1) participants to modify their responses, (2) controlled feedback of group responses from the previous round to give participants an idea of the panel’s aggregated views, and (3) quasi-anonymity [6, 25]. Quasi-anonymity means that the participants were fully anonymous to each other as well as to the whole research team, except for the study coordinator who was responsible for all information exchange. This allowed the participants to express themselves frankly and free of the peer-group pressures and other social dynamics inherent in non-anonymous group discussions. Ethics approval was granted by the University of Edinburgh Law School Research Ethics and Integrity Committee.

Experts were selected by the research team using purposive sampling. They were mostly—but not exclusively—based in the UK. The individuals who agreed to participate in the study described their primary professional responsibilities in their own words (see below), on the basis of which they were categorised as either a researcher, a clinician or a regulator, with some participants claiming primary responsibilities falling under more than one of these three categories, thus reflecting the hybrid nature of their work. In this small-scale piece of research, we did not seek to recruit patients or members of the public, whose responses might also be more fully captured using qualitative interviews.

The study comprised three rounds in order to provide enough opportunity for the participants to modify their responses in light of their participation in the Delphi exercise, as well as to avoid attrition due to too many rounds [5, 9, 26]. Rounds 2 and 3 of the survey included questions about participant’s experiences of the current research culture and instances of best practice; their attitudes to risk-based approaches to regulation; their opinions on how to achieve regulatory efficiency and responsive regulation; their thoughts on public interest and the role of public engagement; and finally how they saw the future of health research regulation.

The study was run via the Bristol Online Survey tool, each round running for 7 weeks and spaced 6–10 weeks apart. Up to 2 additional reminders were sent to experts for each round. No financial incentives were offered for participation.

105 experts (roughly equal numbers of men and women) were invited to participate in the first round, and 29 completed it. Of these, 23 completed round 2, and 20 completed round 3. Of the final 20 participants, 17 were from the UK, 6 were female. As their primary professional responsibilities, 10 of the final participants named ‘research’ activities (in health and in the social sciences), 7 named regulatory activities, 2 clinical duties, and 1 named both research and clinical duties. Of the 9 participants who did not complete all three rounds, 3 were researchers, 1 a regulator, and 5 were hybrid researchers/regulators. Table 1 presents the participants’ characteristics in more detail.

**Table 1:** Participant characteristics (n = 20 in round 3)

Age	
Mean (SD)	48.85 (9.79)
Age range	35–70 years
Gender [n (%)]	
Male	14 (70%)
Country of residence [n (%)]	
UK	17 (85%)
Australia	1 (5%)
Canada	1 (5%)
USA	1 (5%)
Primary professional responsibilities	
Clinical Neurologist, Data Access Consultant, Ethical Review, Health Service Data Protection, Neuroradiologist, Oversight of Primary Care Service Organization, Project Management, Public Health Consultant, Research *(Biomedical Ethics, Biotechnology, Imaging Research, Law* (2)*, Political Science, Population Health Data, Population Health Genetics, Social Psychology, Sociology),* Stakeholder Engagement, Training for REC members	

### Process

Round 1 ran from April to June 2017 and consisted of 14 open questions regarding the research topic and an additional 8 socio-demographic questions. 29 participants contributed a total of 396 responses to the 14 open-ended questions consisting of a total of 26,246 words.

Round 2 was built based on the first-round responses and resulted in 70 7-point Likert scale questions and 5 multiple choice questions organised into 10 sections. It ran from August to October 2017. Participants were encouraged to leave qualitative comments to justify their quantitative response. These 23 participants contributed 615 comments consisting of 15,015 words.

Round 3 ran from November 2017 to January 2018. It consisted of 39 of the 75 questions from round 2 presented together with the mean second-round response for Likert scale questions and the frequency with which multiple choice responses were chosen and the qualitative comments from round 2. 36 questions were omitted in round 3 because sufficient homogeneity in second-round responses was observed. 6 of the 43 questions had slight modifications or additions made to the wording to address concerns about ambiguities that participants raised in round 2. An additional 5 novel qualitative questions were added in round 3 to explore salient themes in more depth. The remaining 20 participants contributed 384 comments consisting of 11,608 words.

### Qualitative Analysis

In addition to the quantitative results generated by the study, as reported in “Public Engagement” section, qualitative data was generated by the study and analysed by the project team. Following Braun and Clark [7], this approach involved:

- Familiarisation with the data—including reading, re-reading and noting initial impressions of the data, first by the whole project team, before sections of the study were allocated to specific team members.
- Coding the data—in this process each team member reviewed the sections for which they were responsible in order to identify portions of the text with labels. At this stage, these labels were as specific or broad as required, with a view to some of the categories being collapsed as the analysis progressed. Some of these codes were based on existing research interests, but a significant proportion emerged inductively from the data.
- Collating codes into broad themes—here codes were arranged by broad themes and any redundancy eliminated, again by the allocated team member.
- Reviewing and revising—this was the most labour-intensive part of the process. Here the themes and codes generated in the primary analysis were cross-checked in detail by a second team member, and then discussed as a group. This ensured that further, cross-cutting themes could be identified and allowed for adjustments to be made to themes and codes, ensuring that nothing of importance was missed.

The seven key themes that were identified in this qualitative analysis are reported upon below in the “Conclusion” section.

## Summary of Quantitative Results

Of the 75 Likert statements rated over rounds 2 and 3, 7 garnered agreement or strong agreement (6 or 7 on a 7-point scale) from more than two-thirds of the participants. Of the 6 multiple choice lists, 4 lists had items selected as most important by more than two-thirds of the participants. The following is a breakdown of these results.

The greatest agreement (80% of the participants selecting 6 or 7) was with the concept that ‘the risk of not conducting research must be managed as assiduously as the risk of conducting research’.

Three of the seven items that reached some consensus had to do with obstacles to optimal health research regulation:

- ‘Inconsistent application of data protection legislation and regulation creates barriers to data access in health research’ (75%);
- ‘Values such as the public interest and public benefit as used in health regulation need further elaboration in practice’ (73.9%);
- ‘Rigid legislation can distract decision-makers from considering the overall intention of legislation.’ (69.6%)

The remaining three items highlight specific ways of improving health research regulation:

- ‘Training on information governance ought to be offered to researchers’ (75%);
- ‘Health research regulation would benefit from the inclusion of best practice examples, which can help researchers, RECs and others understand how principles should be applied’ (73.9%);
- ‘Public engagement activities should involve individuals other than patients.’ (70%)

The 4 lists yielding items selected by more than two-thirds of participants can be seen to supplement these 3 items. ‘Best practice examples’ should be ‘identified’ by ‘regulators’ (85%) and ‘researchers’ (75%), and ‘consensus’ on these examples should be ‘reached’ by ‘regulators’ (90%), ‘professional bodies’ (85%), ‘researchers’ (80%), ‘patients/participants’ (80%), and ‘publics’ (80%). The most important ‘aim of public engagement activities’ in health research was deemed to be ‘allowing the perspectives of persons with experience of a condition or intervention to be heard’ (85%).

‘Conditions most important to expedite review of urgent research’ were deemed to be:

- ‘Clearly defined requirements/expectations of what is required’ (73.9%);
- ‘Protection of research participants from harms’ (73.9%);
- ‘A channel for expedited review for urgent research.’ (73.9%)

### Disagreement

In addition, five areas with significantly divergent views within the participant panel were identified, i.e. at least a quarter of the panel agreed with the statement and at least a quarter disagreed. Three of these were related to the purpose of health research regulation:

- ‘Regulatory frameworks help researchers to focus on delivering public benefits and interests’;
- ‘Health research regulation currently encourages ethical reflection by researchers’;
- ‘Priority is split between delivery of a streamlined regulatory system and attempting to improve the ethical awareness and skill-base of health researchers.’

The panel was similarly split regarding the idea that ‘a system of “trusted researchers” should be developed to allow fast-track approvals’ as well as on whether patients and publics should be involved in the following stages of the health research process: ‘methodology decisions’, ‘drafting grant applications’, and ‘interpreting the results’. As reported below, we explored these last themes more fully with the participants because they represented unexpected results.

## Summary of Qualitative Results

### Regulatory Uncertainty

The responses to the Delphi survey capture a core, recurrent theme that cuts across the regulation of health regulation, namely uncertainty [29, 30]. Uncertainty, of course, can arise in multiple forms in this field; research by its very nature is an uncertain endeavour. Yet, here we found that particular concern was largely expressed about researcher confidence in correctly interpreting, and thus complying with, regulation. That is, the uncertainty was not about research itself, but rather about the rules and regulations that must be observed in order to conduct lawful and ethically acceptable research. We can thus derive two sub-themes from the responses. First, inefficient legal responses to uncertainty [30] mean that those who fall within the rubric of regulatory rules are often left with various difficulties about how to navigate them. Second, some participants reported that the law is creating uncertainty for various ‘partners’ in the health research process, especially for researchers.

Of the forms of uncertainty that might be experienced by stakeholders, one persistent theme that arose from the participants’ responses related to the ways in which regulations and guidance are interpreted, especially where regulatory provisions are cross-cutting or operate in overlapping ways. For example, some respondents offered the view that law can be too generic, leaving researchers uncertain as to whether law even applies to them. An example, consistent with wider commentary [11, 21] is the thorny subject of data protection as it relates to health research. As also reflected in the quantitative data, participants reported confusion around the provisions of the new General Data Protection Regulation (GDPR) with regards to research exemptions and the requisite levels of consent (see Box 1). Discussion of the GDPR also highlighted the challenges which frequently-changing legislation can present for actors in understanding their responsibilities. A tension emerged between, on one hand, suggestions that regulators ought to do more to support sponsors and researchers, and on the other hand, that sponsors ought to take on more responsibility in terms of supporting researchers. Likewise, it was suggested that regulators have a responsibility to be clear about which activities are prohibited and permitted. As shown more fully below, this emphasises a common theme to emerge from this study around the idea and importance of collective responsibility in health research regulation.

The survey also found that researchers often feel uncertain as to whether their proposed project complies with one or more regulatory framework, especially when new forms of research and/or innovation do not seem to fit neatly within a given regulatory framework. New projects may overlap multiple guidance/policies/regulatory spheres: ‘Complex research is often faced with such uncertainty: examples include where research purposes seem to straddle research/clinical boundaries such as in genomics of rare diseases’ (21, researcher).^1^ Further, some participants expressed concern that guidance is easily misinterpreted, especially if a given law is complex: ‘Regulatory frameworks can be pretty hard to understand. Categorisation of research practices within frameworks is a key problem’ (30, researcher). The Delphi results reflected a call for language used in regulations and/or guidance to be clear, accessible and specific. Some respondents reported that language used in regulation could become obsolete if too specifically tied to particular technologies. Equally, participants did recognise the need for technology-specific regulation in certain cases. The challenge is to develop regulatory frameworks that can adapt to reflect more recent uses of words and phrasings often employed in health research environments. This issue emerged as part of a larger call for regulation to be more responsive to new forms of research as well as new challenges, as we discuss further in “Flexibility” section.

Yet, not only did results show uncertainty surrounding the interpretation of, and compliance with, particular rules, but also, more broadly, with general principles of health research regulation. For example, participants expressed difficulties in understanding where the public interest lies in health research regulation. In particular, results highlighted a perceived lack of relevance of abstract legal notions of public interest to health research regulation: ‘Public interest seems too nebulous, residing in case law and judges’ superior consciousness’ (20, clinician/researcher). Uncertainty around legal notions of the public interest can obstruct otherwise beneficial health research. Furthermore, there was doubt about how public interests relate to public engagement: ‘Values such as the public interest and public benefit as used in health regulation need to be specified much better’ (10, researcher). We return to these concepts in “Public Interest” section. For now, the challenge to emerge is two-fold: while clearer conceptualisation of public interest is required, it has also been suggested that public involvement/engagement is required to feed into any future conceptualisation. In other words, these regulatory concepts or approaches do not exist in isolation: they influence and are formed by each other. While this might be a truism in the abstract, it has a tendency to create more uncertainty within regulatory environments in practice, and generates a degree of anxiety about what and who is implicated in such processes.

The ability of stakeholders to appropriately identify and discharge the responsibilities incumbent upon them is reliant at least in part upon the existence of clear legislation within the regulatory setting. However, as we shall see below, legislation is only one means of guiding researchers’ practices (see “Roles and Responsibilities of Key Actors in Shaping the Regulatory Framework” and “Flexibility” sections). Respondents provided examples from across the research spectrum where unclear legislation raises uncertainties around how, and in what circumstances, they ought to act, for example when reporting adverse events. A strong theme to emerge from our engagement with participants was the role of sponsors in this regard. As one participant stated: ‘I can see what the regulations are trying to do but they are very burdensome, prone to overinterpretation by sponsors, this muddies the research and thus does not enable researchers’ (25, researcher). The same respondent argued, ‘Rigid application of regulations by some sponsors risks absolving the researcher of any moral responsibility—certainly does not encourage it—and becomes a tick box process which is dangerous.’ This suggests that attention needs to be paid to bottleneck moments in regulatory timelines; sometimes, upstream actors can significantly delay or otherwise adversely impact effective regulation in ways that are not transparent or obvious to regulators and other stakeholders.

Two final themes that emerged among the responses regarding uncertainty and interpretation were the need for researchers themselves to take individual and collective responsibility for the interpretation of law, but participants also reported the need for support in this interpretive endeavour. It thus seems that there is a balance to be struck here between supporting researchers and asking them to act autonomously. All balancing exercises require a degree of judgement, and in the next section we address one of the more concrete concepts that requires this of stakeholders: proportionality.

### Proportionality

Proportionality in health research regulation has long been a clarion call among many stakeholders, from regulators and regulatees alike [2]. Indeed, its role in delivering effective regulation has taken on the mantle of a self-evident truth in the literature as well as policy and practice circles: who would advocate for a *disproportionate* system? Multiple examples now exist of attempts to incorporate proportionality into regulatory regimes, including the UK’s REC proportionate review service [14], which allows for expedited ethics review if there are ‘no material ethical issues’ in a proposed research project.

However, the reality of attempting to give effect to proportionality in practice is far more nuanced than a simplistic cost/benefit balance might suggest, and this has resulted in subtle distinctions in understanding and experience. For example, the Delphi survey participants reported that while proportionality has increased, the overall complexity of the systems has also increased. There was a sense that this has occurred independent of assessments of actual research risk, and that regulation has increasingly taken on the form of ‘form filling’.

This raises the crucial question: what kind of exercise is the pursuit of proportionality anyway? It can be easy to reduce this to a techno-bureaucratic risk/benefit assessment, but this misses the fact that the search for proportionality is a moral assessment of whether, when and how to proceed in the face of uncertainty. As another participant commented: ‘[c]urrent frameworks distract researchers with process detail which detracts from the quality of the science, delays progress, wastes money and may actually increase the risk to participants’ (25, researcher). Conducting research in a way that contributes to the creation of social value is also itself a moral matter. As other participants commented on regulatory regimes: ‘…bureaucracy and frameworks can stand in the way of creativity and innovation…’ (15, clinician), and also ‘…adding value by subtracting adverse impacts seems scalar but is more complex: it may also limit research directions (e.g. constraints on research involving children)’ (24, regulator). A further participant put it simply thus: ‘The risk of not conducting research is an ethical issue and therefore should be taken into account’ (21, researcher).

There is an understandable tendency when engaging with proportionality to see this as a risk-based assessment. But, as the above responses demonstrate, proportionality is more than mere risk-management. Diverse research protocols involve a wide range of considerations, for example there may be harms to participants or groups that go far beyond the physical, such as identity harms [24]: ‘…risks are not just physical (e.g. for forearm studies). The psychosocial risks of less invasive research should not be underplayed’. In contrast, some outcomes—such as imminent death in emergency situations—can lead to tolerance and acceptability of very high-risk interventions [12]. Delphi participants picked up on these points in various ways. For example, ‘[m]any actors (e.g. NHS regulators) tend to be risk averse and this can prevent useful research where the risk is less than perceived’ (19, regulator), while at the other end of the spectrum it was also commented that ‘… [w]ell established simple interventions can involve very considerable risk. Researching complexity can be difficult for biomedical research focused committees to understand, as indeed can straightforward qualitative research; I would not want to see more obstacles placed in the way of such work’ (15, clinician).

More presciently, the following important point was made about the generalisable nature of all research activity: ‘It is less likely that the procedures applied to research participants will get substantially more complex; rather than complexity lies in what happens to the data derived from them; where it goes, how it is used now and in future’ (5, researcher). This highlights crucial questions about the stages in the research lifecycle at which assessments of proportionality are carried out. As we have argued elsewhere, the timeframes involved in realising the social value of human health research are often extremely attenuated, and the range of actors involved in these processes are diverse and often unconnected [13]. This has implications for regulatory pathways and the—perhaps repeated—role for proportionality therein. The reporting of adverse events was frequently seen by Delphi participants to be a downstream disproportionate activity:

…the definitions of adverse events result in vast numbers of daily events being classed as reportable with result that trial gets bogged down in documenting the utter unrelated trivia that are common in patients with some disorders and unrelated to the drug to the neglect of collecting complete and high quality baseline and outcome data on which the reliability of the results depend (25, researcher).

As to ways to navigate increased complexity—and to manage proportionality effectively—some important ideas emerged from the research. One participant advocated ‘regulators etc. becoming helpers and guiding processes to make approval more feasible. Whilst having a proportionate outlook’ (27, clinician). This has resonance with our own argument for an increased role for regulatory stewardship to help guide researchers through regulatory landscapes [19]. Others called for ‘networked governance’ whereby, among other things, ‘…regulatory agencies in health (broadly understood) would need to engage more with academics and charities, and to look to utilise a broader range of expertise in designing and implementing governance strategies and mechanisms’ (5, researcher).

This points beyond proportionality to matters of how to assess the public interests at stake, and we now turn to this theme, drawing on the findings from “Regulatory Uncertainty” section about the utility of appeals to public interest and its seemingly eternal amorphous nature.

### Public Interest

The Delphi participants recognised that one of the key challenges of realising values such as ‘public benefit’ and ‘the public interest’ is the need to take into account various interests of diverse publics and other stakeholders. It was noted that these interests might run contrary to one another or appear to be mutually exclusive (19, regulator). A particular concern was the very practical issue of how to capture the range of opinions engaged in health research regulation. For example, one participant noted that ‘…the loudest voices aren’t the ones that we need to listen to’ (6, regulator). Some commented on the risk that small but vocal groups might dominate the discourse on health research (with the corollary implication that there would be other voices that would not be heard) (20, clinician/researcher). Together, these highlighted to us the relationship between the role of the public interest, as a concept, and the potential role of ‘good’ public engagement to inform and interact with this concept. We discuss this topic further in “Public Engagement” section.

Some were of the view, more generally, that health research is very (perhaps overly) focused on the individual participant. It was suggested that this ‘…was understandable but not always helpful and often to the detriment of other legitimate interests’. When considering this tension others urged that ‘…there should generally be more attention to the public/social level of benefits and harms, in addition to the individual level’ (16, researcher).

To find a way forward, participants looked to transparency—both of process and of values—as part of the solution. One participant noted that giving an acceptable account of *how* different views, and in particular minority views, were taken into account in health research is crucial (24, regulator). Another elaborated on this theme, suggesting that research is based on trust, which will be ‘…best promoted by involving all those with legitimate interests’ (2, regulator). Comments included that it was ‘important for researchers to be able to explain and justify their work in lay terms’ (19, regulator), acknowledging that there had been some, albeit slow, improvement here. This has resonance with the findings explained in “Regulatory Uncertainty” section that suggest a need for all stakeholders to engage in the collective responsibility of giving effect to regulatory concepts and tools, such as the public interest.

As noted above, in the report of the quantitative findings from the study, there was strong agreement by the participants in relation to the statement that: *values such as the public interest and public benefit as used in health regulation need further elaboration in practice.* This was also reflected in the qualitative data where the need for researchers to be able to articulate the public benefit and public interest in their work was addressed specifically by a number of participants (17, regulator and 21, researcher). One participant’s experience was that ‘…researchers are often poor at being able to articulate the public interests and benefits in their work, making their application seem riskier’ (17, regulator). Others suggested that this might be because the benefits of research could sometimes be taken for granted (16, researcher), hence the focus instead on preventing harm. A specific example given of where this might be difficult related to commercial players in health research. The view was expressed that the new GDPR would bring the ‘articulation of public interest for data processing into sharper focus’ (21, researcher). Another concern was that these concepts were too nebulous. Some called for greater clarity, ‘…in both a conceptual (in relation to other key values and concepts) and a situated sense (within the context of particular research initiatives)’ (24, regulator), again echoing the findings in “Regulatory Uncertainty” section.

Achieving clarity around public benefit and public interests was acknowledged to be a challenge (24 regulator and 16 researcher). However, when looking forward to possible futures for health research regulation, participants argued for more emphasis on this, and for health research to support these values. As one participant noted: ‘We need the key voices of health…being publically supportive of research and good examples of the value it brings to individuals’ (13, regulator). Examples given of these ‘key voices’ included the Chief Medical Advisor to the UK government and the CSO (Chief Scientist Office) in Scotland. As well as this call for further articulation of the value of research, it was recognised that governance strategies and mechanisms would be required to support this work.

### Public Engagement

As highlighted in “Public Interest” section, the Delphi study generated evidence of the importance of appreciating the mutually complementary roles of public interest and public engagement. This is a direct reflection of the growing recognition that patients and members of the public can play a role in various, but not all, areas of medical (and other science) research [15, 22]. This role is usually described as public engagement or involvement in research. Many of the Delphi participants responded to our questions about public engagement by discussing public-patient-involvement (PPI) or public involvement (PI) instead. However, despite this interchangeable use, one respondent commented that ‘engagement (not involvement) is for everyone. The two things aren’t the same by the way’ (5, researcher). Although both are more active than awareness or understanding, only involvement suggests participation in the research process itself.

Underlying this inconsistency in terminology is a lack of shared understanding about who counts as ‘the public’. Answers referring to ‘broader publics (8, researcher)’, ‘harder to reach people and groupings’ (19, regulator) and ‘patients and potential patients’ (24, regulator) all acknowledge that the public is not a homogeneous entity. Despite the often stated argument that ‘we will all be patients one day’ (25, researcher), the public cannot be easily equated to (potential) patients: as one respondent argued, patients and potential patients have ‘complex priorities, [that are] sometimes in conflict’ (24, regulator). Although patient involvement is becoming a more routine part of medical research, respondents described how ‘the same token patients’ attending meetings ‘tend to be white, retired and middle class’ (6, regulator). This a problem across many kinds of engagement initiatives that rely on voluntary participation. Individuals who have the resources to participate in such activities are usually educated and articulate [8], leading to the danger that the needs and priorities of other groups are not recognised. Future research needs to go beyond generic questions about public engagement and look at the possibilities for participation in specific contexts, including identifying which groups are missing out. The ‘difficult to reach’ can quickly become the ‘easy to ignore’.

It was suggested that there might be a role for ‘deliberative research’ methods here, as well as room for more public engagement in multiple research settings and at different stages of the research enterprise. Successful public engagement was defined as ‘facilitating two-way understanding’ (5, researcher) between researchers and publics. Ideally, such conversations give members of the public a better understanding of the benefits of research, how it is practised and some of the constraints involved. At the same time, dialogue with non-researchers also allows researchers to develop a better understanding of what (some) publics want from research, especially the types of research they deem important. Some respondents did fall back onto ‘deficit’ models [10] to argue that, because of a lack of technical knowledge, members of the public should not be asked for advice about research funding priorities. However, several other participants in the Delphi study made the opposite point that listening to a wider range of viewpoints and incorporating them into research policy or designs can improve research. However, this is a time-consuming process, not least because it requires trust to develop between researchers or clinicians and their publics [20], and so the pursuit of meaningful public engagement adds to both the direct and transactional costs of research projects. Many funders now expect research projects to include an element of public engagement, but our data suggests that this still goes against established ways of working within medical research. Not only will researchers have to develop new ways of writing grants and academic articles, but as one respondent suggested: ‘we need to be given the institutional license to do this and to make mistakes and try new things. Otherwise it’s too risky to our careers’ (5, researcher). If success can no longer be understood solely within a scientific model of ‘hard evidence’, then new ways of conceptualising of what it means to do good research must be developed. One such means is co-production, which forms the focus of the next section.

### Roles and Responsibilities of Key Actors in Shaping the Regulatory Framework

Co-production and collaboration at different junctures of the health research endeavour emerged as important themes within the survey, implying that a variety of stakeholders have a role to play in shaping the regulatory framework. From the perspective of public and patient involvement, this is in keeping with a wider shift towards increased stakeholder engagement (see “Public Engagement” section above). The findings also demonstrate acknowledgement that the development of regulatory approaches ought to involve partnership between regulators and researchers. This relationship was described as essential in order to achieve regulatory efficiency and in the identification of, and reaching consensus on, instances of best practice. One respondent explained that such collaboration between regulators and experienced researchers could provide the context-sensitivity necessary to develop best practice (24, regulator), an important function of best practice which has been considered elsewhere [28].

While there appeared to be a general appetite for the inclusion of stakeholders, interesting nuances emerged around which stakeholders ought to be involved in different processes, and what weighting their preferences ought to be given (16 and 30, researchers). For example, some respondents suggested that potential roles for publics and patients in shaping research were context-dependent, as described in “Public Engagement” section.

One way in which roles and responsibilities, permitted and prohibited activities, as well as instances of best practice can be communicated to researchers, is through training. Respondents were surveyed on their attitudes towards the prospect of requiring accreditation of researchers in order to grant them ‘trusted’ or ‘accredited’ status which could lead to fast-track oversight of research applications. However, this was met with a mixed reaction; although some participants thought accreditation could improve flexibility and responsiveness, a considerable number expressed scepticism, arguing that accreditation might create a power imbalance between accredited and non-accredited researchers and lead to possible lapses in oversight. Would an accredited researcher be audited? Would it lead to an ‘old boys’ network? Others felt that accreditation could be misplaced, perhaps serving only to gauge the researcher’s character or track record to date, rather than the appropriateness of the research design and research system as a whole. Consequently, a few participants advocated a mixed accreditation system for certain areas of research where both researchers and sponsors are accredited and subject to regular review and audit. This did not extend, however, to Phase 1 clinical trials or research involving vulnerable persons.

### Flexibility

There was concern that the health research regulatory system as it currently functions is too rigid; what participants desired was a flexible system, built less around command-driven rules and more on proportionate but clear high-level principles that are facilitative of research (5, researcher and 6,). Just as respondents advocated for greater certainty in regulation, so too did they call for regulation that permitted them a wide regulatory space in which to operate. Certainty and flexibility in regulation were not seen as mutually exclusive principles. As also noted in the quantitative results, the Delphi survey participants emphasised the importance of systemic approaches in regulation, and the need for a robust architecture that supports the various component parts working in harmony.

For example, some participants felt that sponsors of research projects (e.g. universities, NHS trusts, pharmaceutical companies) take a strict interpretative approach to regulation, which encourages a disproportionate, ‘tick-box process.’ This is illustrated most starkly in the requirements for reporting adverse events in clinical trials of an investigational medicinal product (CTIMPs). As noted above, requirements to report minor adverse events that are not necessarily relevant to the research aims can mean that critical tasks such as the collection of complete and high-quality baseline and outcome data—on which the reliability of the results depend—get neglected. This ‘mismatch’ between what clinical researchers see as contextually serious, and what the regulations define as necessary to report, can lead to clogged regulatory spaces and research projects tangled in a regulatory mire [30]; see also [18]. Participants voiced a preference that each step of the regulatory process be more carefully managed, with a view to bringing the research project to fruition—and then sustained through completion—as ethically and efficiently as possible.

To overcome rigidity and the sway of a tick-box approach, some participants saw flexibility as a core component of ‘principles-based regulation’, which is seen as a beneficial, but not problem-free, regulatory approach in health research. Principles-based regulation involves regulators outlining principles to achieve regulatory objectives and values, and regulatees then devising their own system to serve the principles [4, 27]. Within such an approach, regulation could become more adaptive to emerging technologies and encourage researchers and sponsors to engage in ongoing conversations with regulators on ways to manage risks and achieve defined regulatory objectives. Others argued that flexibility works best in a hybrid regulatory system comprised of both principles and rules. As one participant put it, ‘principles-based approaches offer greater flexibility but [the principles] need [to be] interpreted. They work best when they are translated into rules-based guidelines that are revised and updated at sensible intervals. You need both [principles and rules] working in tandem’ (5, researcher). Similarly, and reflecting the observation that certainty and flexibility need not be mutually exclusive, another participant stressed that they liked ‘the idea of flexible principles, but this relies on training and time to reach consensus. Clear rules are more easily applied when resources are limited’ (15, clinician). This would suggest a regulatory design that allows for both certainty and flexibility, depending on factors such as resource constraints and stakeholder demand. However, others felt that too much emphasis on rules as a means of providing clarity would not unclog the regulatory space in health research. Instead, these participants stressed the need for proportionate and flexible approaches, and for regulators to encourage researchers and sponsors to adapt approaches that work best for them, albeit within an architecture that provides structure and ethical bounds of what is considered safe and responsible behaviour.

Together, these narratives simultaneously underlined the potential difficulties of reaching consensus in our pluralistic society, as well as the crucial importance of cooperation and co-production in research design and delivery.

### Looking to the Future

In addition to the principal broad themes detailed above, a number of other issues were raised by participants in the study. These are worthy of attention because they speak pointedly to the time and political climate in which this study was conducted.

A particular issue that was raised was the role of artificial intelligence (AI) or machine-learning in health research and healthcare. Examples of the possible uses of AI in health research include its application in identifying patterns within or between large datasets, and in helping to identify suitable participants for clinical studies [23]. Respondents to this study recognised the potential benefits of AI to health research regulation itself, for example to enhance regulators’ realistic assessment of research risks. Nevertheless, it was also recognised that the use of AI itself carries ‘very substantial risks’ (15, clinician) arising from, for example, the incorporation of ‘deliberate or unconscious biases in the design of the tasks, functions and priorities of machine learning systems’ (5, researcher), a lack of transparency and difficulties locating ethical responsibility for decisions reached on the basis of AI analyses. As such it was noted that the use of AI in healthcare should also itself be the *subject* of research to better understand the incidence and management of these risks. Within the bigger picture of health research regulatory environments, AI is a notable example of the disruptive impact of new technology on health research regulation, but is merely the latest illustration of the on-going uncertainty of the regulatory enterprise itself. This feature will never be eliminated, but our study suggests ways in which uncertainty and opportunity can be better accommodated within adaptive regulatory structures as part of cooperative partnerships between stakeholders.

The importance of regulators establishing regulatory connections, particularly with regards to supporting cross-border collaboration, was raised by participants. This was discussed with specific reference to the challenges of working across different legislative jurisdictions, for example in multi-centre trials. This issue has become particularly acute in the context of the disruptive effects of the United Kingdom’s potential departure from the European Union. Yet again, the overarching concern amongst participants was uncertainty, this time about what the UK regulatory landscape would look like post-Brexit. Some participants suggested that Brexit could provide an opportunity to revisit what ‘right regulation’ (30, researcher) might look like. Meanwhile, others expressed concerns that it could instead bring ‘change, but not necessarily for the better’ (16, researcher), by inviting chaos and ‘a race to the bottom’ (2, regulator) in terms of regulatory standards. Participants reported that ‘another worrying aspect of Brexit’ (17, regulator) was the threat it posed to much-needed international collaboration between research regulators. Indeed, in disentangling its health research regulation from European legal frameworks, while also seeking future regulatory alignment, the UK is at risk of creating ‘liminal hotspots’ in a number of fields, including the regulation of clinical trials and licensing of medicines [17]. These hotspots will be characterised not only by the present uncertainty engendered by transition towards an unclear goal, but also the future threat of ossified replacement laws that fail to evolve in step with the rest of Europe. This would represent the antithesis of the clear call for readily-interpreted and responsive regulation emerging from this research.

These examples are particularly pertinent given the emphasis that our participants placed upon clarity and consistency in the application of regulations and the value of regulatory systems that are both proportionate and sufficiently flexible to be responsive. These goals are not necessarily mutually exclusive objectives as some might assume. Underpinning this research is a sense across all stakeholders of a common purpose and a willingness to try. Law and regulation must not stifle that commitment with excessive rigidity or the prospect of undue sanction. Compliance culture no longer accurately reflects the needs and expectations of researchers or regulators, nor does it necessarily produce the best research. Embracing uncertainty—both as a human practice and a regulatory objective—may represent the brighter future for health research.

## Conclusion

This Delphi study was undertaken in order to provide an interdisciplinary and crosscutting analysis of health research regulation as it is experienced by regulators, researchers and other expert stakeholders. The results outlined above provide five important new insights into the operation of current UK and European regulatory environments. A key theme emerging from participants’ responses is a call for greater collaboration, and even co-production, of research regulation by various stakeholders, in the service of achieving a responsive, flexible and proportionate response to the challenges and critical value that health research offers. Future research should complement this perspective by investigating the responses of other key stakeholders—including patients and members of various publics—to this call.

First, our participants argued that lawmakers and regulators should continue to steer health research regulation away from a strict, prescriptive rules-based regime, and towards flexibility and a principles-based regime allowing researchers to co-produce their own systems with other stakeholders and to serve core principles. Such flexible approaches enable regulation to be more adaptive to emerging technologies, and also encourage researchers and sponsors to engage in ongoing conversations with regulators in order to manage risks and achieve defined regulatory objectives. These conversations, in turn, allow for the practice of systems of regulatory stewardship, where regulators work with researchers and sponsors to work through the different stages of the regulatory process efficiently, ethically and legally.

Second, many respondents also highlighted the uncertainty that researchers experience in interpreting and, therefore, properly complying with regulations. In such cases, a key issue is the need to strike a balance between attributing responsibility for the interpretation of law, and the provision of support in carrying out that interpretation. Although practices of regulatory stewardship may be one means of addressing this uncertainty, our findings suggest that a further exploration of both effective means of collaborative modes of regulation and the pursuit of a fresh understanding of proportionality might inform us how to better meet this need.

Third, the call for greater mobilisation of collaboration and co-production implies a diffusion of responsibility whereby regulation becomes the common concern of a range of actors—including researchers, regulators, publics and research sponsors. At the same time, it is clear that these different groups cannot all participate in all stages of the regulatory process to the same degree. For example, the principle of wider public (and other stakeholder) engagement and involvement in regulation is broadly accepted. However, our results demonstrate that debates are still taking place about: what form this participation should take; which individuals and groups should be encouraged to participate; at what stage of the research cycle it is practical for them to do so; and how much weighting the preferences of each of these stakeholders ought to be given. Answers to these questions will vary depending both on the type of research in question and on the specific stakeholder group in question. This emphasises the importance not only of the *who* question in engagement, but also the *when*. If engagement is to be meaningful, its undertaking must be timely with respect to both when engagement takes place, and what is then done with the findings to improve regulatory processes.

Fourth, this study also provides a situated account of what public interest means to stakeholders in regulation. Achieving a better understanding of the public interest is an important element of the guiding principles underlying collective responsibility. Like public engagement, public interest is always context-specific. More specifically, participants pointed to the ways in which techniques of public engagement can feed into our understanding of how the public interest is accomplished in practice. The results emphasised the need for transparency when trying to establish its meaning in a particular context, for example through the articulation of the value of research and the benefits that this might deliver to specific populations or groups. When public interest and public engagement are taken together, the value and vagueness of appeals to public interest become less contentious, and the public interest becomes potentially more effective as a regulatory device. Thus, meanings of public interest must be captured in context, and further life must be breathed into them by active demonstration of how public interest in any given situation is properly to be realised.

Finally, proportionality was seen by our participants as a crucial element of flexible regulation. While the value of proportionality in regulation is currently well-recognised, these results demonstrate that it is much more than a mere risk-management tool. Importantly, it involves an ethical assessment of values and risks at sake at multiple junctures in the trajectory of conducting research: from study design to feedback of results. As with other aspects of regulation, a range of actors are therefore implicated in the role that proportionality must play. Upstream light-touch ethics review can be thwarted by downstream disproportionate regulatory requirements, such as required policies on incidental findings where such findings are highly unlikely to appear. This study therefore represents a call to reimagine proportionality as a regulatory device not merely as a means of risk-reduction, but more as a focus for the ethical assessment of the values and risks at stake at multiple junctures in the trajectory of conducting research. To do so equips actors with a tool to live with—and through—the constant uncertainties that typify the domain of human health research.

## Electronic supplementary material

Below is the link to the electronic supplementary material.

Supplementary material 1 (DOCX 98 kb)

## Footnotes

1 Quotes are labelled with a participant’s number (to maintain anonymity) and their professional role (see “Methods” section).

## References

[1] Academy of Medical Sciences. (2006). *Personal data for public good: Using health information in medical research*. Available at: https://acmedsci.ac.uk/policy/policy-projects/personal-data. Accessed 10 July 2019.

[2] Academy of Medical Sciences. (2011). *A new pathway for the regulation and governance of health research*. Available at: https://acmedsci.ac.uk/policy/policy-projects/a-new-pathway-for-the-regulation-and-governance-of-health-research. Accessed 10 July 2019.

[3] Academy of Medical Sciences. (2016). *Regulation and governance of health research: Five years on*. Available at: https://wellcome.ac.uk/sites/default/files/regulation-and-governance-of-health-research.pdf. Accessed 10 July 2019.

[4] Baldwin R, Cave M, Lodge M (2011). Understanding regulation: Theory, strategy, and practice.

[5] Beech B (1997). Studying the future: A Delphi survey of how multi-disciplinary clinical staff view the likely development of two community mental health centres over the course of the next two years. Journal of Advanced Nursing.

[6] Birko S, Dove ES, Özdemir V (2015). A Delphi technology foresight study: Mapping social construction of scientific evidence on metagenomics tests for water safety. PLoS ONE.

[7] Braun V, Clarke V (2006). Using thematic analysis in psychology. Qualitative Research in Psychology.

[8] Brett J, Staniszewska S, Mockford C, Herron-Marx S, Hughes J, Tysall C, Suleman R (2014). Mapping the impact of patient and public involvement on health and social care research: A systematic review. Health Expectations.

[9] Delbecq AL, Van de Ven AH, Gustafson DH (1975). Group techniques for program planning: A guide to nominal group and Delphi processes.

[10] Dickson, D. (2005). ‘The case for a ‘deficit model’ of science communication’ (editorial). Available at: https://www.scidev.net/global/communication/editorials/the-case-for-a-deficit-model-of-science-communic.html. Accessed 10 July 2019.

[11] Dove ES (2018). The EU general data protection regulation: Implications for international scientific research in the digital era. Journal of Law, Medicine & Ethics.

[12] Ganguli-Mitra, A., & Sethi, N. (2016). *Conducting research in the context of global health emergencies: Identifying key ethical and governance issues*. Background Report, Nuffield Council on Bioethics. Available at: http://nuffieldbioethics.org/wp-content/uploads/Research-in-global-health-emergencies-background-paper.pdf. Accessed 10 July 2019.

[13] Ganguli-Mitra A, Dove ES, Laurie G, Taylor-Alexander S (2017). Reconfiguring social value in health research through the lens of liminality. Bioethics.

[14] HRA. (2018). *Home/about us/committees and services/research ethics service and research ethics committees/developing the REC proportionate review service*. Available at: https://www.hra.nhs.uk/about-us/committees-and-services/res-and-recs/developing-rec-proportionate-review-service/. Accessed 10 July 2019.

[15] INVOLVE. (2018). About us. Available at http://www.invo.org.uk/about-involve. Accessed 10 July 2019.

[16] Laurie G (2017). Liminality and the limits of law in health research regulation: What are we missing in the spaces in-between?. Medical Law Review.

[17] Laurie G (2018). How do we make sense of chaos? Navigating health research regulation through the liminality of the Brexit process. Medical Law International.

[18] Laurie G, Harmon S, Cloatre E, Pickersgill M (2015). Through the thicket and across the divide: Successfully navigating the regulatory landscape in life sciences research. Knowledge, technology and law.

[19] Laurie G, Dove ES, Ganguli-Mitra A, Fletcher I, McMillan C, Sethi N, Sorbie A (2018). Charting regulatory stewardship in health research: Making the invisible visible. Cambridge Quarterly of Healthcare Ethics.

[20] Martin GP, Finn R (2011). Patients as team members: Opportunities, challenges and paradoxes of including patients in multi-professional healthcare teams. Sociology of Health & Illness.

[21] McCall B (2018). What does the GDPR mean for the medical community?. The Lancet.

[22] NCCPE. (2018). *What is public engagement*? Available at: http://www.publicengagement.ac.uk/about-engagement. Accessed 10 July 2019.

[23] Nuffield Council on Bioethics. (2018). *Bioethics briefing note: Artificial intelligence (AI) in healthcare and research.* Available at: http://nuffieldbioethics.org/wp-content/uploads/Artificial-Intelligence-AI-in-healthcare-and-research.pdf. Accessed 10 July 7 2019.

[24] Postan E (2016). Defining ourselves: Personal bioinformation as a tool of narrative self-conception. Journal of Bioethical Inquiry.

[25] Rietjens JA, Sudore RL, Connolly M, van Delden JJ, Drickamer MA, Droger M, van der Heide A, Heyland DK, Houttekier D, Janssen DJ, Orsi L (2017). Definition and recommendations for advance care planning: An international consensus supported by the European Association for Palliative Care. The lancet Oncology.

[26] Rowe G, Wright G (1999). The Delphi technique as a forecasting tool: Issues and analysis. International Journal of Forecasting.

[27] Sethi N (2015). Reimagining regulatory approaches: On the essential role of principles in health research. SCRIPTed.

[28] Sethi N (2018). Research and global health emergencies: On the essential role of best practice. Public Health Ethics.

[29] Sethi, N., Dove, E. S., McMillan, C., Laurie, G., Fletcher, I., Ganguli-Mitra, A., Postan, E., & Sorbie, A. (2018). *Liminal spaces workshop: Regulating for uncertainty*. 1–2 February, 2018, Wellcome, London. Available at: https://www.blogs.law.ed.ac.uk/liminalspaces/wp-content/uploads/sites/12/2018/03/Liminal-Spaces-2018-Workshop-Report-1.pdf. Accessed 10 July 2019.

[30] Taylor-Alexander S, Dove ES, Fletcher I, Ganguli-Mitra A, McMillan C, Laurie G (2016). Beyond regulatory compression: Confronting the liminal spaces of health research regulation. Law, Innovation and Technology.

[31] Vibert F (2014). The new regulatory space: Reframing democratic governance.

