# Supplementary material for: Co-production and Managing Uncertainty in Health Research Regulation: A Delphi Study
Source: Health Care Anal. 2019 Aug 31;28(2):99–120. doi: 10.1007/s10728-019-00383-9 (PMC7210237; doi:10.1007/s10728-019-00383-9)
Supplement: Supplementary file 1 — Supplementary material 1 (DOCX 98 kb) [file 10728_2019_383_MOESM1_ESM.docx]

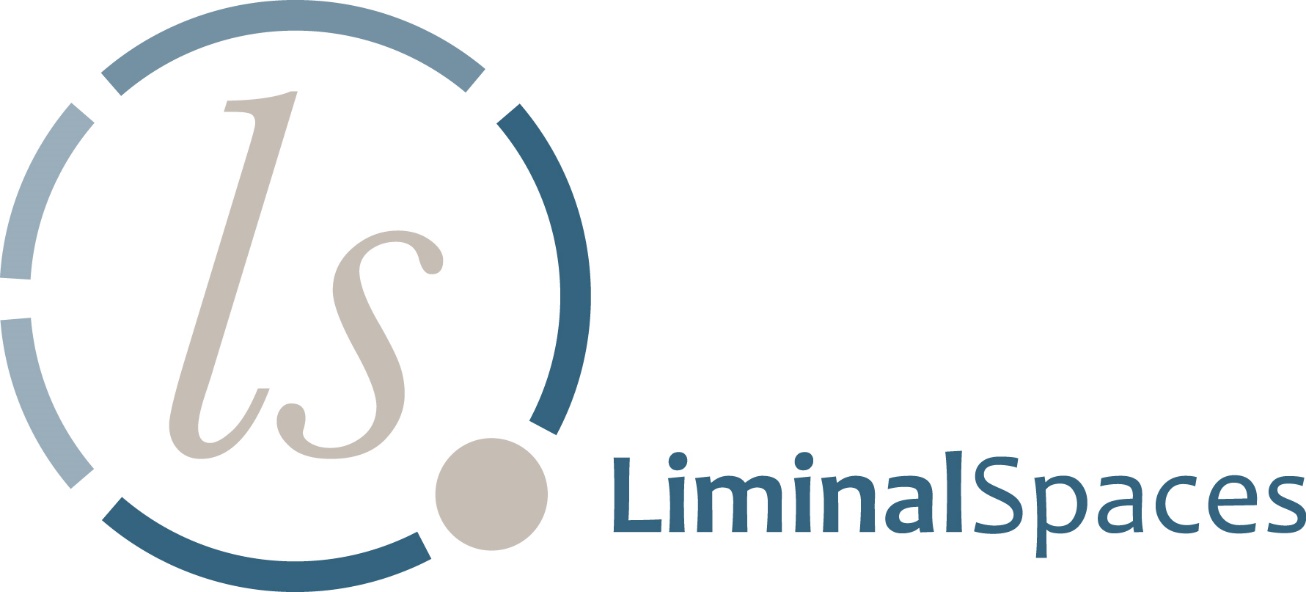


Improving Health Research Regulation: Better by Co-Design?

Methodology and Survey Questions Used in a 3-Round Delphi Survey

February 1^st^, 2018

BRIEF OVERVIEW

The key goal of this research was to analyse the impacts regulation can have on health research according to the perceptions of leading experts. For this purpose, a classic three-round Delphi survey was conducted between April 2017 and January 2018.

The first round consisted of 14 open-ended questions focusing on the three broad themes of: a) “evaluating existing regulatory frameworks”, b) regulating for uncertainty, and c) public engagement; as well as a few socio-demographic questions. The questions of the second round were based on the responses to the first-round open-ended questions. The third round consisted of: 1) questions from round 2 that did not show consensus within the panel of experts together with aggregated and anonymized quantitative and qualitative second-round results; and 2) a few questions derived from second-round comments.

A total of 105 persons were invited to participate, 29 of whom completed the first round. 23/29 participated in the second round, and 20/23 completed all three rounds of the study. This final group consisted of 6 women and 14 men, with ages ranging from 35 to 70, with a median age of 46.5. 17 of the participants were from the UK, 2 from North America and 1 from Australia. 10/20 identified their primary occupation as professors or university-affiliated researchers.

METHODOLOGY

The Delphi technique was used to promote a conversation within a panel of experts regarding the impacts regulation can have on health research. The Delphi technique has been widely used as a means to identify issues of interest in fields where “appropriate historical/economic/technical data [is not available] and thus where some form of human judgmental input is necessary”^[[1]](#footnote-1)^ and is characterized by quasi-anonymity, asynchronicity, iterative questionnaires with statistical analysis and controlled feedback of expert group results^[[2]](#footnote-2)^.

**Ethical considerations**

Quasi-anonymity is a crucial ethical consideration in the context of a Delphi study. Panelists’ lack of knowledge as to the source of each individual response guarantees that the personalities of the respondents do not play a role in influencing others’ behavior. Views that in a face-to-face setting might be considered unpopular or risky to voice are more likely to come up. Moreover, the confidentiality of responses allows the respondents the freedom to change their opinion in subsequent rounds rather than having to defend a locked-in position that has their name attached to it^[[3]](#footnote-3)^. However, true anonymity is impossible to achieve as the Delphi study monitor knows the origin of individual responses. Thus, quasi-anonymity was guaranteed by preserving the confidentiality of individual views and omitting any potentially identifying features from inter-round results fed back to the group as well as from the final report. Quasi-anonymity means that while the individual responses were not completely anonymous to the research team, they were completely anonymous to the rest of the respondents^[[4]](#footnote-4)^.

The principle of asynchronicity is the freedom given panelists to respond when and how is more convenient for them. Finally, the controlled feedback component of the study requires that the group results are reported back to the respondents in as unbiased a manner as possible.

**Number of rounds**

The number of rounds was pre-determined at 3. The procedure originally designed by RAND in the 1950s recommended four rounds, but this has been criticized by many researchers in the last decades, because the fourth round is often plagued by poor response rates thereby compromising the integrity of the results. Many researchers have since suggested two to three rounds^[[5]](#footnote-5)^. In keeping with recommendations in the Delphi literature, a >70% response rate in each round was ensured in order to maintain rigour^[[6]](#footnote-6)^.

The study was conducted between April 2017 and January 2018. The questionnaire was administered in English using the Bristol Online Surveys (BOS) platform. Panelists were invited to each round via email.

**Round 1**

Round 1 queried respondents using 14 open-ended questions regarding issues associated with existing regulatory frameworks, regulating for uncertainty, and public engagement to be rated in subsequent rounds as well as 8 socio-demographic questions.

The data generated during round 1 was clustered into thematic groups, reflecting the participants’ views in the themes thus created. The statements to be used as items in round 2 were kept as close to the originals as possible.

**Round 2**

The second round questionnaire was created by the researchers in light of the first-round answers. A list of statements which participants were invited to rate on 7-point Likert scale was elaborated. There was a total of 70 statements that were rated on 7-point Likert scales, and 5 multiple choice questions. Following the rating scales for each item, space was provided where respondents were encouraged to make explicit the assumptions their ratings were based on as well as to add any other free text comments. Mean scores for each item were calculated in order to present to the panelists in the third round.

**Round 3**

The third round questionnaire consisted of 39 of the items from round 2, with 6 whose wording was slightly modified to address ambiguities pointed out by the panelists. The questions were complemented by a presentation of the aggregate group responses, thus allowing the respondents a chance to respond once again after being exposed to the group opinion. All qualitative comments identified for each issue in round 2, were made explicit to the panel, keeping as always all text fully anonymized. This has been suggested to increase the likelihood of change due to informational influences as opposed to purely normative ones^[[7]](#footnote-7)^.

36 items from round 2 were not included in round 3, given that the second round results displayed sufficient consensus among the panel. 5 further open-ended questions were added as a result of participant comments. Qualitative comments from respondents, including responses to second-round comments as well as novel issues, were again encouraged.

QUESTIONNAIRE (ROUNDS 2 & 3 COMBINED)

Experiences of the current research culture

## Regulatory frameworks help researchers to focus on delivering public benefits and interests.

## Regulatory processes can take so long that projects become unviable. (Never – Always)

## Regulatory frameworks add value to research and innovation by minimising adverse impact on participants. (Never – Always)

## Consistent regulatory procedures promote effective health research.

## The benefits of health research regulation are currently well understood by the general public.

--- Round 2 ONLY ---

## Complex research is faced with uncertainty around complying with regulatory frameworks.

## Current health research regulation is driven by: (Compliance only – Researcher conscience only)

## Instances of best practices

## Select which of the following groups should be involved in identifying and reaching consensus on best practice examples.

|  | Identify | Reach Consensus | Neither |
| --- | --- | --- | --- |
| Regulators |  |  |  |
| Professional bodies |  |  |  |
| Researchers |  |  |  |
| Funders |  |  |  |
| Patients/participants |  |  |  |
| Publics |  |  |  |

## Examples of bad practice should be offered to illustrate how guidance should not be interpreted.

--- Round 2 ONLY ---

## Health research regulation would benefit from the inclusion of best practice examples (in guidance documents, etc.), which can help researchers, RECs and others understand how principles should be applied.

## Health research regulators should be responsible for identifying and promoting best practice examples.

## Professional bodies, such as the GMC or the Academy of Medical Sciences, should include best practice examples within their guidance.

## Select which of the following examples constitute effective approaches to health research regulation:

Public Benefit and Privacy Panel in Scotland

EU Clinical Trials Regulation

The Integrated Research Application System (IRAS)

Health Research Authority Approvals Process

The Caldicott Principles

## Risk-based approaches

## The risk of not conducting research must be managed as assiduously as the risk of conducting research.

## Regulation should be proportionate to the level of risk in a research study, i.e. minimal risk research should be subject to less regulation.

## Health research regulation should adopt adaptive approaches to risk management, that is, flexible governance arrangements when the risks are low.

--- Round 2 ONLY ---

## Publics have a far less risk averse approach to research compared to regulators.

## Regulations should shift in focus from facilitation of research to protection of participants and the public as the complexity of the research increases.

## Towards regulatory efficiency

## Over time there have been improvements in the proportionality of health research regulation overall.

## Inconsistent application of data protection legislation and regulation creates barriers to data access in health research.

## The process of reporting adverse events in clinical trials is rather: (A burden – A benefit)

--- Round 2 ONLY ---

## To promote regulatory efficiency regulators and researchers need to work in partnership to understand the challenges of their respective roles.

## Better collaboration between health research regulators across boundaries (either in terms of regulatory objects* or geography) is key to regulatory efficiency.

## Please choose the five (5) conditions you consider to be most important to expedite review of urgent research:

## Cost efficiency

## Minimal regulatory burden for researchers

## Consistency / harmonisation of health research requirements / standards

## Flexibility and open mindedness of health research regulators

## Clearly defined requirements / expectations of what is required

## Accountability of individual researchers for proper research conduct

## Adequately resourced and supported regulatory bodies

## Protection of research participant from harms

## Protection of researchers from harms

## A channel for expedited review for urgent research

## Involvement of publics

## Responsive regulation

## Clear rules are more important than flexible principles.

## Rule-based approaches are required to define clear parameters between permitted and prohibited activities.

## Regulation should avoid the use of technology-specific language.

--- Round 2 ONLY ---

## Principle-based approaches are preferable to rule-based approaches in regulating health research.

## It is more important for regulation to be flexible and adaptive than rigid and prescriptive.

## It is possible for regulation to be both flexible and clear.

## Rigid legislation can:

## Stifle innovation

## Lead to regulatory lapse

## Distract decision-makers from considering the overall intention of legislation

## Regulatory Responsibilities

## Sponsors and R&D offices represent a bottleneck in attaining regulatory approval.

## Regulators should act as stewards to guide researchers through the entire regulatory approval process.

## A system of “trusted accredited researchers” should be developed to allow fast-track approvals.

## The following ought to be included in a system of trusted accredited researchers:

## Training on research approval mechanisms

## Training on information governance

## Training on ethical reflection and discussion

## Certification by a recognised authority

## Registration by a recognised professional authority

## Enhanced continuing professional development opportunities

--- Round 2 ONLY ---

## Delivering effective research regulation is the responsibility of all parties involved in health research.

## Regulators should work in partnership with researchers in designing effective regulation.

## Regulatory systems should allow for safe spaces for researchers to explore new approaches to health research leading to novel research outcomes or products.

## Public benefit and public interest

## A key challenge of realising values such as public benefit and public interest is the need to take into account the various interests of diverse publics.

## Health research regulation focuses too much on possible risks and harms to individuals and not enough on public interests and benefits.

## Uncertainty around legal notions of the public interest can obstruct otherwise beneficial health research.

--- Round 2 ONLY ---

## Values such as the public interest and public benefit as used in health regulation need further elaboration in practice.

## There should be more clarity around how decisions about public benefit and public interest are made by regulators.

## Fear of possible legal liability causes researchers to not feel safe when trying to judge where the public interest lies in health research regulation.

## Public engagement

## The principal use of public engagement should be for setting research funding priorities.

## Public engagement activities should involve individuals other than patients.

## It is difficult to ensure that views received from public engagement exercises are representative.

## What are the three (3) most important aims of public engagement activities?

## Allowing the perspectives of marginalised groups to be heard

## Allowing the perspectives of persons with experience of a condition or intervention to be heard

## Defining areas that need future research

## Educating members of the public about the conduct of research

## Ensuring more ethical conduct of research

## Increasing transparency in the spending of public money

## Determining the most appropriate ways of discussing particular issues (incl. vocabulary for example)

--- Round 2 ONLY ---

## There should be involvement by patients and publics at the following stages of the health research process

### **Identifying research needs**

### **Drafting a research protocol**

### **Methodology decisions**

### **Drafting grant applications**

### **Applying for ethical approval**

### **Recruiting participants (when applicable)**

### **Collecting data**

### **Interpreting the results**

### **Knowledge translation**

### **Evaluating research impact**

## Patients and/or their carers have too much influence in research compared to other groups.

## Professionalism

## Health research regulation currently encourages ethical reflection by researchers.

## Adherence to current health research regulation processes is likely to improve researchers’ ability to recognise and handle ethical issues in health research.

## Between delivery of a streamlined regulatory system (1) and attempting to improve the ethical awareness and skills-base (7) of health researchers, which is more of a priority?

## A system of “trusted accredited researchers” would improve the ethical acceptability of health research in the eyes of the public.

## Health research should be governed by: (Self-regulation – Governmental regulation)

## In Round 2 responses to this question, the prospect of ‘co-produced regulation’ was raised. Participants in this round are invited to comment on this below, both as to the possible meaning of this term, whether it is desirable, and what it might mean in practice.

--- Round 2 ONLY ---

## Regulators should act as a ‘sounding board’ for researchers to explore new approaches to health research.

## Possible Futures

## ‘Brexit’ creates an opportunity for the UK to redesign health research regulation that it is more effective.

## Please choose the two (2) most plausible future scenarios (within the next ten years) in health research regulation.

## Increasingly restrictive regulation will lead to less research being conducted.

## Regulatory consolidation will occur, where different regulators are joined together to cover multiple areas.

## There will be better coordination among regulatory bodies.

## Regulation will become more adaptive to emerging areas of research.

## Regulation will fail to adequately capture participant-led research.

## An increasing amount of information in consent forms and participant information sheets will be required by regulation.

## Proportionality will become a common feature in regulation.

## Please tell us about the future scenario in health research regulation (within the next ten years) that you would like to see.

## What would need to change in the regulatory environment, if at all, to bring about your desired scenario?

## Machine learning and artificial intelligence are being hailed as the next technologies to change healthcare provision. What do you think will be the main ethical and legal issues to arise from such developments? Please comment on how adequate current regulatory structures are for addressing such possible issues.

## If you have any further comments regarding this third round questionnaire, please leave them here:

QUESTIONNAIRE (ROUND 1)

**A. Evaluating existing regulatory frameworks**

**In this section, we invite you to consider existing regulatory frameworks and to describe your experiences of working in (or being subjected to) one or more research areas, and how regulation impacts on those areas. The common theme here is to ask questions about regulatory frameworks that are viewed as successful, neutral, or problematic – and why.**

1. Can you describe your experiences (good, bad, confusing, etc.) of current regulatory frameworks in health research? We suggest you offer three examples to demonstrate these experiences.
2. What examples of successes or improvements in regulatory frameworks can you describe, and why do you consider them successes or improvements? We suggest you offer three examples.
3. What regulatory approaches (e.g. rules-based, principles-based/best practices) and forms of regulation (e.g. legislation, professional guidance, ethical codes) do you think work particularly effectively in different domains of health research, and why?

**B. Regulating for uncertainty: novel or complex research areas**

**In this section, we invite you to consider areas of research that are ‘novel’ or ‘complex’. By ‘novel’ we mean fields of research or research questions that break new ground in their area of enquiry. By ‘complex’ we mean research projects that are particularly challenging in their design, for example, because they straddle two or more regulatory domains, and/or they require engagement with a wide set of actors, and/or the research project itself raises challenging social or ethical issues. The common theme here is to ask questions about research that tests the limits or parameters of regulatory regimes.**

1. In what ways do you think regulation does (or can) enable researchers to better navigate novel or complex research areas?
2. In what ways do you think regulation does not (or cannot) enable researchers to better navigate novel or complex research areas?
3. What conditions do you believe are required to promote effective regulation between different sectors of scientific enquiry, especially to improve regulatory efficiency for emerging health research? Please describe what effective means to you, if relevant.
4. What conditions do you believe are required to respond adequately to novel or complex research areas, including from the perspective of the role of law, regulators and other actors in the regulatory domain?
5. What undesired consequences can regulatory approaches to health research have? How can these be mitigated?
6. Please describe two to four future scenarios of health research regulation that seem most plausible to you. Set your scenarios ten years from now, i.e., 2027.

**C. Public engagement – upstream and downstream**

**In this section, we invite you to consider opportunities for engagement by publics (i.e. different communities of people) in policymaking and regulation, and to consider values such as ‘public interest’ and ‘public benefit’. ‘Publics’ might refer to groups within the general public of citizens, or groups of researchers, or other groups interested in, or affected by, health research and its regulation.**

**The common theme here is to ask questions about engagement both *upstream* (i.e. at the early stages of research design, or of law and policymaking) and *downstream* (i.e. after policy or regulation has already been developed and/or implemented).**

**By ‘public interest’, we mean common interests that the general public has cause to accept. By ‘public benefit’, we mean a purpose or activity that benefits the public in general, or a sufficient section of the public (e.g. better health of the population).**

1. What role, if any, should engagement with publics (incl. other health research stakeholder groups) have to help inform developments in laws and policies affecting health research regulation? Which publics might play a role?
2. What role, if any, should engagement with publics (incl. other health research stakeholder groups) have across different points in the research lifecycle? Which publics might play a role and at which stages? Please explain your reasoning and assumptions in detail.
3. Are values such as ‘public interest’ and ‘public benefit’ effectively reflected in health research regulation? Please give examples to explain your views.
4. How might values such as ‘public interest’ and ‘public benefit’ be better reflected in health research regulation going forward, if at all? Please give examples to explain your views.
5. Are there any other aspects of regulatory approaches to emerging health research not addressed in the above questions that you would like to see discussed in future rounds?
6. How likely to you believe yourself to be able to revise your views as a result of participating in the current three-round Delphi study? Please use a scale of 0 to 10, where 0 indicates that you believe your views to be rock-solid, and 10 indicates as much openness as possible to change your mind on the topic.

1. Rowe, G., Wright, G., “The Delphi technique as a forecasting tool: issues and analysis”, International Journal of Forecasting 15 (1999) 353–375. [↑](#footnote-ref-1)
2. Goodman, C., 1987. The Delphi technique: a critique. Journal of Advanced Nursing 12, 729–734. [↑](#footnote-ref-2)
3. Delbecq, A.L., Van de Ven, A. & Gustafson, D. (1975) *Group Techniques for Program Planning: A Guide to Normal Group and Delphi Processes*. Scott, Foreman and Company, Glenview, Illinois. [↑](#footnote-ref-3)
4. Keeney, S., Hasson, F., McKenna, H. (2011) The Delphi Technique in Nursing and Health Research, Wiley 2010;Blackwell,Oxford, UK. [↑](#footnote-ref-4)
5. Green, B., Jones, M., Hughes, D. & Williams, A. (1999) Applying the Delphi technique in a study of GPs information requirement. *Health and Social Care in the Community* 7(3), 198–205. [↑](#footnote-ref-5)
6. Sumsion, T. (1998) The Delphi technique: an adaptive research tool. *British Journal of Occupational Therapy* 61(4), 153–156. [↑](#footnote-ref-6)
7. Rowe G (1992). Perspectives on expertise in the aggregation of judgments. In: Wright G, Bolger F, editors. Expertise and decision support. New York : Plenum Press. p. 155–80. [↑](#footnote-ref-7)
